# Crystal structure of {*N* ^1^,*N* ^3^-bis­[(1-benzyl-1*H*-1,2,3-triazol-4-yl)methyl­idene]-2,2-di­methyl­propane-1,3-di­amine}bis­(thio­cyanato-κ*N*)iron(II)

**DOI:** 10.1107/S2056989020012608

**Published:** 2020-09-22

**Authors:** Kateryna Znovjyak, Maksym Seredyuk, Sergey O. Malinkin, Sergiu Shova, Lutfullo Soliev

**Affiliations:** a Department of Chemistry, Taras Shevchenko National University of Kyiv, Volodymyrska Street 64, Kyiv, 01601, Ukraine; b Department of Inorganic Polymers, "Petru Poni" Institute of Macromolecular, Chemistry, Romanian Academy of Science, Aleea Grigore Ghica Voda 41-A, Iasi, 700487, Romania; c Department of General and Inorganic Chemistry, Faculty of Chemistry, Tajik State Pedagogical University, Rudaki 121, 734003 Dushanbe, Tajikistan

**Keywords:** iron(II) complex, thio­cyanate complex, high spin state, trigonal distortion, crystal structure

## Abstract

The unit cell of the title compound, [Fe^II^(NCS)_2_(C_25_H_28_N_8_)], consists of two charge-neutral complex mol­ecules related by an inversion centre. In the complex mol­ecule, the tetra­dentate ligand *N* ^1^,*N* ^3^-bis­[(1-benzyl-1*H*-1,2,3-triazol-4-yl)methyl­ene]-2,2-di­methyl­propane-1,3-di­amine coordinates to the Fe^II^ ion through the N atoms of the 1,2,3-triazole moieties and aldimine groups. Two thio­cyanate anions, coordinating through their N atoms, complete the coordination sphere of the central ion. In the crystal, neighbouring mol­ecules are linked through weak C—H⋯π, C—H⋯S and C—H⋯N inter­actions into a two-dimensional network extending parallel to (011). The inter­molecular contacts were qu­anti­fied using Hirshfeld surface analysis and two-dimensional fingerprint plots, revealing the relative contributions of the contacts to the crystal packing to be H⋯H (35.2%), H⋯C/C⋯H (26.4%), H⋯S/S⋯H (19.3%) and H⋯N/N⋯H (13.9%).

## Abstract 2

The title compound shows a *cis*-arrangement of the thio­cyanate anions, while the coordination polyhedron around the iron(II) atom is close to a trigonal prism.

## Chemical context   

Coordination complexes of 3*d* transition metals represent a large class of potentially applicable materials exhibiting catalytic (Strotmeyer *et al.*, 2003 ▸), magnetic (Pavlishchuk *et al.*, 2010 ▸) and spin-switching functionalities (Gütlich & Goodwin, 2004 ▸) with easily detectable and exploitable variations of physical properties (Gural’skiy *et al.*, 2012 ▸; Suleimanov *et al.*, 2015 ▸).

Iron(II) complexes based on Schiff bases derived from N-substituted 1,2,3-triazole aldehydes represent an inter­esting class of coordination compounds exhibiting spin-state switching between low- and high-spin states in different temperature regions (Hagiwara *et al.*, 2014 ▸, 2016 ▸, 2020 ▸; Hora & Hagiwara, 2017 ▸). In charge-neutral mononuclear complexes of this kind described so far, the thio­cyanate anions occupy the axial position of the coordination sphere and thus are in a *trans*-configuration (Hagiwara & Okada, 2016 ▸; Hagiwara *et al.*, 2017 ▸).

Having ongoing inter­est in functional 3*d* metal complexes formed by polydentate ligands (Seredyuk *et al.*, 2006 ▸, 2007 ▸, 2011 ▸, 2015 ▸, 2016 ▸; Seredyuk, 2012 ▸; Valverde-Muñoz *et al.*, 2020 ▸), we report here the synthesis and crystal structure of a new high-spin Fe^II^ complex based on the tetra­dentate ligand *N* ^1^,*N* ^3^-bis­[(1-benzyl-1*H*-1,2,3-triazol-4-yl)methyl­ene]-2,2-di­methyl­propane-1,3-di­amine with thio­cyanate anions arranged in a *cis*-configuration.
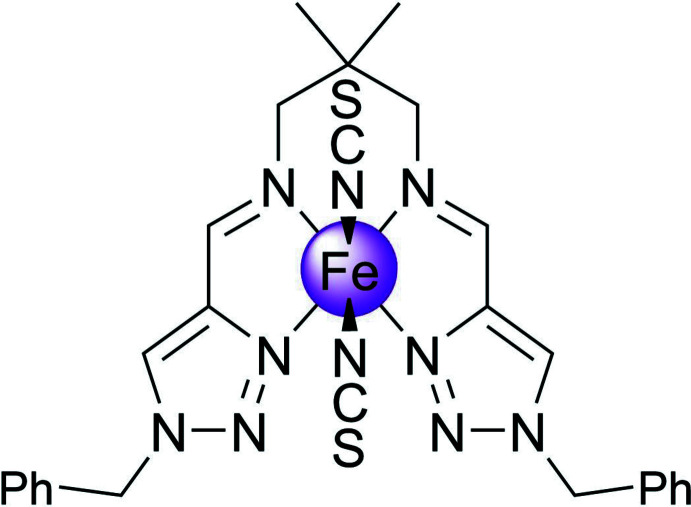


## Structural commentary   

**Figure 1:**
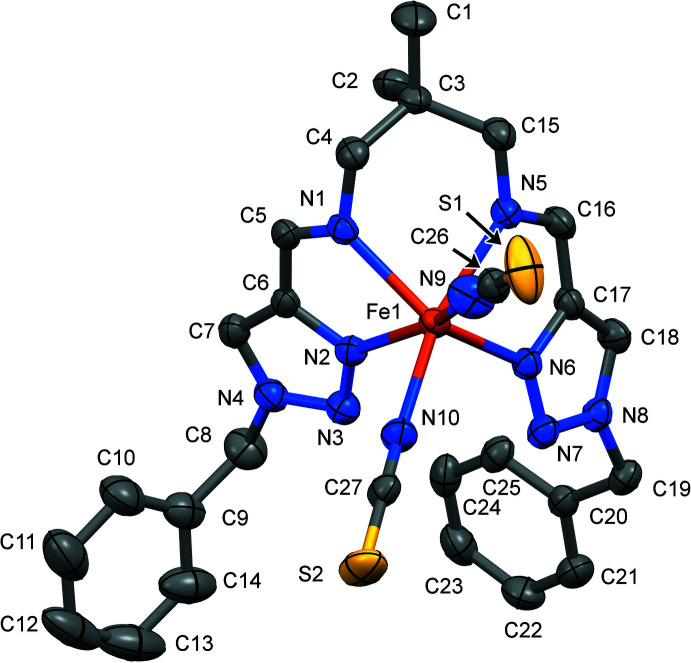
The mol­ecular structure of the title compound with displacement ellipsoids drawn at the 50% probability level. H atoms have been omitted for clarity.

The Fe^II^ ion of the title complex has a distorted trigonal–prismatic N_6_ coordination environment formed by four N atoms of the tetra­dentate Schiff-base ligand and two NCS^−^ counter-ions (Fig. 1 ▸). The average bond length <Fe—N> = 2.19 (9) Å is typical for high-spin complexes with an [FeN_6_] chromophore (Gütlich & Goodwin, 2004 ▸). The N—Fe—N angle between the *cis*-aligned thio­cyanate N atoms is 87.58 (9)°. The average trigonal distortion parameters Σ = Σ_1_^12^(|90 − *φ*_*i*_|), where *φ*_*i*_ is the angle N—Fe—N′ (Drew *et al.*, 1995 ▸), and Θ = Σ_1_^24^(|60 − *θ*_*i*_|), where *θ*_*i*_ is the angle generated by superposition of two opposite faces of an octa­hedron (Chang *et al.*, 1990 ▸), are 453.2 and 149.38°, respectively. These values reveal a great deviation of the coordination environment from an ideal octa­hedron (where Σ = Θ = 0), and are significantly larger than those of similar [FeN_6_] high-spin *trans*-complexes (Hagiwara *et al.*, 2017 ▸). With the aid of continuous shape measure (CShM), the closest shape of a coordination polyhedron and its distortion can be determined numerically (Kershaw Cook *et al.*, 2015 ▸). The calculated CShM value relative to the ideal *O_h_* symmetry for an octa­hedron is 6.285, while it is 4.008 relative to the ideal *D*_3*h*_ symmetry for a trigonal prism. Hence, the polyhedron is closer to the latter shape; however, it is notably distorted (for the ideal polyhedron CShM = 0). The volume of the [FeN_6_] coordination polyhedron is 12.4 Å^3^.

## Supra­molecular features   

**Figure 2:**
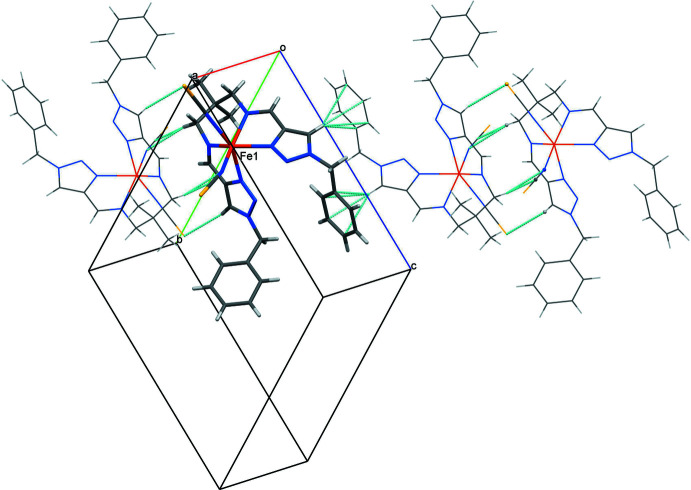
Weak hydrogen bonding (cyan dashed lines), resulting in the formation of chains in the packing.

Neighbouring complex mol­ecules form dimers through double weak contacts C18—H18*B*⋯*Cg* ^i^ of 3.330 (3) Å (*Cg* corres­ponds to the centroid of the C20–C25 phenyl ring; symmetry codes refer to Table 1 ▸). The CH group of one of the triazole rings forms a weak hydrogen bond C7—H7⋯S1^ii^ [3.755 (3) Å] with a thio­cyanate anion. This, together with the C4—H4*B*⋯C27^ii^ and C4—H4*B*⋯N10^ii^ inter­actions [3.709 (3) and 3.617 (3) Å] involving the C≡N group of the anion, links the dimers into a supra­molecular chain propagating parallel to [01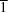] (Fig. 2 ▸). These chains are weakly bound through double contacts between the benzyl groups and the thio­cyanate anions [C21—H21⋯C27^iii^ = 3.603 (3) Å] and triazole groups [C19—H19*A*⋯N7^iii^ = 3.311 (3) Å] of neighbouring complex mol­ecules, forming a two-dimensional supra­molecular array extending parallel to (011).

## Hirshfeld surface and 2D fingerprint plots   

**Figure 3:**
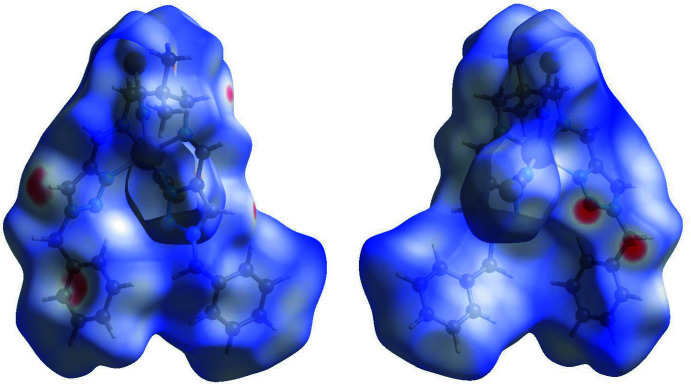
Two projections of *d*_norm_ mapped on Hirshfeld surfaces, showing the inter­molecular inter­actions within the mol­ecule. Red areas represent contacts shorter than the sum of the van der Waals radii, while blue areas represent regions where contacts are larger than the sum of van der Waals radii, and white areas are zones close to the sum of van der Waals radii.

**Figure 4:**
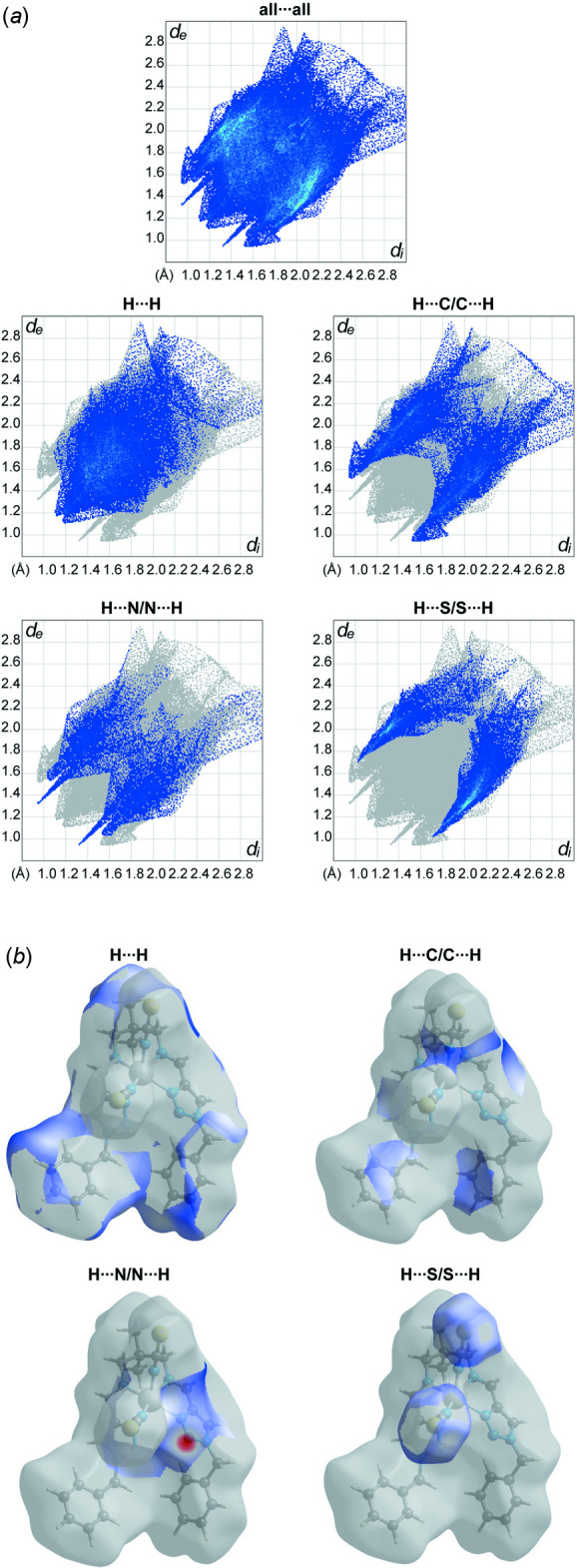
(*a*) The overall two-dimensional fingerprint plot and those decomposed into specified inter­actions. (*b*) Hirshfeld surface representations with the function *d*_norm_ plotted onto the surface for the different inter­actions.

Hirshfeld surface analysis was performed and the associated two-dimensional fingerprint plots were generated using *Crystal Explorer* (Turner *et al.*, 2018 ▸), with a standard resolution of the three-dimensional *d*_norm_ surfaces plotted over a fixed colour scale of −0.2801 (red) to 1.8236 (blue) a.u. The pale-red spots symbolize short contacts and negative *d*_norm_ values on the surface correspond to the inter­actions described above. The overall two-dimensional fingerprint plot is illus­trated in Fig. 3 ▸. The Hirshfeld surfaces mapped over *d*_norm_ are shown for the H⋯H, H⋯C/C⋯H, H⋯S/S⋯H, and H⋯N/N⋯H contacts, and the two-dimensional fingerprint plots are presented in Fig. 4 ▸, associated with their relative contributions to the Hirshfeld surface. At 35.2%, the largest contribution to the overall crystal packing is from H⋯H inter­actions, which are located in the middle region of the fingerprint plot. H⋯C/C⋯H contacts contribute 26.4%, and the H⋯S/S⋯H contacts contribute 19.3% to the Hirshfeld surface, both resulting in a pair of characteristic wings. The H⋯N/N⋯H contacts, represented by a pair of sharp spikes in the fingerprint plot, make a 13.9% contribution to the Hirshfeld surface.

## Database survey   

A search of the Cambridge Structural Database (CSD 2020, update of May 2020; Groom *et al.*, 2016 ▸) revealed four similar Fe^II^ thio­cyanate complexes, derivatives of a 1,3-di­amino­propanes and *N*-substituted 1,2,3-triazole aldehydes, *viz*. DURXEV, ADAQUU, ADAREF and solvatomorphs ADAROP and ADARUV (Hagiwara *et al.*, 2017 ▸; Hagiwara & Okada, 2016 ▸). These complexes show hysteretic spin crossover with the Fe—N distances in the range 1.931–1.959 Å for the low-spin state and 2.154–2.169 Å for the high-spin state of the Fe^II^ ions. The reported pseudo-trigonal–prismatic complexes with an [FeN_6_] chromophore are formed by structurally hindered rigid hexa­dentate ligands favoring a trigonal–prismatic environment of the central Fe^II^ ion in the low- or high-spin state: CABLOH (Voloshin *et al.*, 2001 ▸), BUNSAF (El Hajj *et al.*, 2009 ▸), OWIHAE (Seredyuk *et al.*, 2011 ▸), OTANOO (Stock *et al.*, 2016 ▸). For comparison purposes, Table 2 ▸ collates the distortion parameters Σ, Θ and CShM for the latter complexes.

## Synthesis and crystallization   

The ligand of the title compound was obtained *in situ* by condensation of 1 eq. of 2,2-dimethyl-1,3-propanedi­amine with 2.2 eq. of 1-benzyl-1*H*-1,2,3-triazole-4-carbaldehyde in boiling methanol over 5 min and subsequent reaction with 1 eq. of [Fe(py)_4_(NCS)_2_] dissolved in a minimum amount of boiling methanol with a minimum amount of ascorbic acid. The formed yellow solution was slowly cooled to ambient temperature. The formed orange crystals were subsequently filtered off. Elemental analysis calculated (%) for C_27_H_28_FeN_10_S_2_: C, 52.94; H, 4.61; N, 22.87; S, 10.47; found: C, 52.88; H, 4.37; N, 22.40; S, 10.35. IR *v*KBr (cm^−1^): 1615 (C=N), 2071, 2115 (NCS).

## Refinement   

Crystal data, data collection and structure refinement details are summarized in Table 3 ▸. H atoms were placed in calculated positions using idealized geometries, with C—H = 0.96–0.97 Å for methyl­ene and methyl groups and 0.93 Å for aromatic H atoms, and refined using a riding model with *U*_iso_(H) = 1.2–1.5*U*_eq_(C).

## Supplementary Material

Crystal structure: contains datablock(s) I. DOI: 10.1107/S2056989020012608/wm5580sup1.cif

Structure factors: contains datablock(s) I. DOI: 10.1107/S2056989020012608/wm5580Isup2.hkl

Supporting information file. DOI: 10.1107/S2056989020012608/wm5580Isup3.cdx

CCDC reference: 2032292

Additional supporting information:  crystallographic information; 3D view; checkCIF report

## Figures and Tables

**Table 1:** Hydrogen-bond geometry (Å, °) *Cg* is the centroid of the C20–C25 ring.

*D*—H⋯*A*	*D*—H	H⋯*A*	*D*⋯*A*	*D*—H⋯*A*
C18—H18⋯*Cg* ^i^	0.93	2.42	3.330 (3)	167
C19—H19*A*⋯N7^ii^	0.97	2.38	3.311 (3)	162
C21—H21⋯C27^ii^	0.93	2.89	3.603 (3)	134
C7—H7⋯S1^iii^	0.93	2.87	3.755 (3)	159
C4—H4*B*⋯N10^iii^	0.97	2.69	3.617 (3)	160
C4—H4*B*⋯C27^iii^	0.97	2.75	3.709 (3)	171

Symmetry codes: (i) 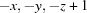; (ii) 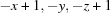; (iii) 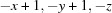.

**Table 2:** Comparison of the distortion parameters for indicated Fe^II^ complexes Parameters for OTANOO averaged over five independent complex cations.

Compound	<Fe–N> (Å)	Σ (°)	Θ (°)	CShM (*D*_3*h*_)
Title compound	2.186	453.2	149.38	4.008
CABLOH	1.899	725.74	178.16	0.525
BUNSAF	2.218	703.65	201.07	1.887
OWIHAE	2.202	894.48	206.57	0.602
OTANOO	2.191	697.3	183.24	1.098

**Table 3:** Experimental details

Crystal data	
Chemical formula	[Fe(NCS)_2_(C_25_H_28_N_8_)]
*M*_r_	612.56
Crystal system, space group	Triclinic, *P* 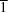
Temperature (K)	250
*a*, *b*, *c* (Å)	8.9656 (5), 12.5060 (6), 14.2311 (7)
α, β, γ (°)	67.552 (5), 85.106 (4), 84.087 (4)
*V* (Å^3^)	1465.06 (14)
*Z*	2
Radiation type	Mo *K*α
μ (mm^−1^)	0.69
Crystal size (mm)	0.4 × 0.2 × 0.2

Data collection	
Diffractometer	Rigaku Oxford Diffraction Xcalibur, Eos
Absorption correction	Multi-scan (*CrysAlis PRO*; Rigaku OD, 2018 ▸)
*T*_min_, *T*_max_	0.911, 1.000
No. of measured, independent and observed [*I* > 2σ(*I*)] reflections	10677, 5175, 4416
*R*_int_	0.018
(sin θ/λ)_max_ (Å^−1^)	0.595

Refinement	
*R*[*F* ^2^ > 2σ(*F* ^2^)], *wR*(*F* ^2^), *S*	0.037, 0.082, 1.03
No. of reflections	5175
No. of parameters	391
H-atom treatment	Only H-atom displacement parameters refined
Δρ_max_, Δρ_min_ (e Å^−3^)	0.62, −0.59

Computer programs: *CrysAlis PRO* (Rigaku OD, 2018 ▸), *olex2.solve* (Bourhis *et al.*, 2015 ▸), *SHELXL2018/3* (Sheldrick, 2015 ▸) and *OLEX2* (Dolomanov *et al.*, 2009 ▸).

## supplementary crystallographic information

### Crystal data

**Table:**

[Fe(NCS)_2_(C_25_H_28_N_8_)]	*Z* = 2
*M**_r_* = 612.56	*F*(000) = 636
Triclinic, *P*1	*D*_x_ = 1.389 Mg m^−^^3^
*a* = 8.9656 (5) Å	Mo *K*α radiation, λ = 0.71073 Å
*b* = 12.5060 (6) Å	Cell parameters from 4582 reflections
*c* = 14.2311 (7) Å	θ = 1.6–28.8°
α = 67.552 (5)°	µ = 0.69 mm^−^^1^
β = 85.106 (4)°	*T* = 250 K
γ = 84.087 (4)°	Plate, orange
*V* = 1465.06 (14) Å^3^	0.4 × 0.2 × 0.2 mm

### Data collection

**Table:**

Rigaku Oxford Diffraction Xcalibur, Eos diffractometer	5175 independent reflections
Radiation source: fine-focus sealed X-ray tube, Enhance (Mo) X-ray Source	4416 reflections with *I* > 2σ(*I*)
Graphite monochromator	*R*_int_ = 0.018
Detector resolution: 16.1593 pixels mm^-1^	θ_max_ = 25.0°, θ_min_ = 1.6°
ω scans	*h* = −10→9
Absorption correction: multi-scan (CrysAlisPro; Rigaku OD, 2018)	*k* = −14→13
*T*_min_ = 0.911, *T*_max_ = 1.000	*l* = −16→16
10677 measured reflections	

### Refinement

**Table:**

Refinement on *F*^2^	0 restraints
Least-squares matrix: full	Hydrogen site location: inferred from neighbouring sites
*R*[*F*^2^ > 2σ(*F*^2^)] = 0.037	Only H-atom displacement parameters refined
*wR*(*F*^2^) = 0.082	*w* = 1/[σ^2^(*F*_o_^2^) + (0.0224*P*)^2^ + 1.1951*P*] where *P* = (*F*_o_^2^ + 2*F*_c_^2^)/3
*S* = 1.03	(Δ/σ)_max_ < 0.001
5175 reflections	Δρ_max_ = 0.62 e Å^−^^3^
391 parameters	Δρ_min_ = −0.59 e Å^−^^3^

### Special details

**Table:**

Geometry. All esds (except the esd in the dihedral angle between two l.s. planes) are estimated using the full covariance matrix. The cell esds are taken into account individually in the estimation of esds in distances, angles and torsion angles; correlations between esds in cell parameters are only used when they are defined by crystal symmetry. An approximate (isotropic) treatment of cell esds is used for estimating esds involving l.s. planes.

### Fractional atomic coordinates and isotropic or equivalent isotropic displacement parameters (Å^2^)

**Table:**

	*x*	*y*	*z*	*U*_iso_*/*U*_eq_	
Fe1	0.46967 (4)	0.25650 (3)	0.15072 (2)	0.02848 (10)	
S1	0.83937 (11)	0.13105 (7)	−0.06302 (8)	0.0749 (3)	
S2	0.93628 (8)	0.30840 (7)	0.26836 (6)	0.0597 (2)	
N1	0.3705 (2)	0.40653 (16)	0.02733 (14)	0.0288 (4)	
N2	0.3331 (2)	0.37376 (16)	0.22397 (14)	0.0307 (4)	
N3	0.3079 (2)	0.37587 (17)	0.31494 (15)	0.0371 (5)	
N4	0.2416 (2)	0.48269 (17)	0.30170 (15)	0.0357 (5)	
N5	0.3032 (2)	0.17554 (16)	0.09218 (14)	0.0310 (4)	
N6	0.3792 (2)	0.11839 (15)	0.28389 (14)	0.0296 (4)	
N7	0.3924 (2)	0.08273 (16)	0.38237 (15)	0.0331 (5)	
N8	0.2832 (2)	0.01043 (15)	0.42600 (14)	0.0306 (4)	
N9	0.6253 (3)	0.2060 (2)	0.05480 (19)	0.0528 (6)	
N10	0.6514 (2)	0.28477 (17)	0.21860 (16)	0.0385 (5)	
C1	0.2253 (4)	0.3438 (3)	−0.1867 (2)	0.0555 (8)	
H1A	0.193892	0.423117	−0.225831	0.056 (9)*	
H1B	0.152987	0.294205	−0.190475	0.070 (10)*	
H1C	0.321299	0.323624	−0.213362	0.070 (10)*	
C2	0.0854 (3)	0.3642 (2)	−0.0346 (2)	0.0419 (6)	
H2A	0.091918	0.352941	0.035621	0.049 (8)*	
H2B	0.010603	0.317602	−0.040245	0.053 (8)*	
H2C	0.058264	0.444495	−0.073289	0.058 (9)*	
C3	0.2375 (3)	0.3284 (2)	−0.07558 (17)	0.0341 (5)	
C4	0.3598 (3)	0.4059 (2)	−0.07427 (17)	0.0346 (6)	
H4A	0.455840	0.377726	−0.096377	0.029 (6)*	
H4B	0.336486	0.484535	−0.121707	0.038 (7)*	
C5	0.2984 (3)	0.48861 (19)	0.04771 (18)	0.0307 (5)	
H5	0.256242	0.553702	−0.003344	0.033 (6)*	
C6	0.2840 (2)	0.47738 (18)	0.15301 (17)	0.0290 (5)	
C7	0.2245 (3)	0.5470 (2)	0.20293 (19)	0.0351 (6)	
H7	0.181423	0.622568	0.174611	0.031 (6)*	
C8	0.1953 (3)	0.5106 (3)	0.3915 (2)	0.0493 (7)	
H8A	0.190249	0.438538	0.450396	0.066 (9)*	
H8B	0.094871	0.548892	0.382656	0.067 (10)*	
C9	0.2961 (3)	0.5868 (2)	0.4139 (2)	0.0463 (7)	
C10	0.3637 (4)	0.6766 (3)	0.3392 (3)	0.0616 (8)	
H10	0.351491	0.691169	0.271088	0.064 (9)*	
C11	0.4509 (4)	0.7463 (3)	0.3655 (4)	0.0805 (11)	
H11	0.497489	0.806878	0.314888	0.080 (12)*	
C12	0.4680 (4)	0.7252 (4)	0.4664 (4)	0.0851 (13)	
H12	0.527762	0.770575	0.483973	0.106 (14)*	
C13	0.3976 (4)	0.6382 (5)	0.5402 (4)	0.0874 (13)	
H13	0.406323	0.625738	0.608290	0.104 (14)*	
C14	0.3135 (4)	0.5684 (4)	0.5147 (3)	0.0666 (10)	
H14	0.267676	0.507861	0.565894	0.104 (15)*	
C15	0.2819 (3)	0.2003 (2)	−0.01518 (18)	0.0373 (6)	
H15A	0.204471	0.153918	−0.019696	0.042 (7)*	
H15B	0.374371	0.177465	−0.045658	0.038 (7)*	
C16	0.2287 (3)	0.0988 (2)	0.15956 (18)	0.0364 (6)	
H16	0.155892	0.062534	0.142359	0.048 (8)*	
C17	0.2622 (3)	0.07019 (19)	0.26449 (18)	0.0314 (5)	
C18	0.2011 (3)	0.0009 (2)	0.35577 (18)	0.0357 (6)	
H18	0.119557	−0.043570	0.366906	0.042 (7)*	
C19	0.2629 (3)	−0.0405 (2)	0.53669 (17)	0.0348 (6)	
H19A	0.359759	−0.070943	0.565445	0.038 (7)*	
H19B	0.200022	−0.104871	0.555534	0.019 (5)*	
C20	0.1924 (2)	0.04492 (19)	0.58170 (17)	0.0306 (5)	
C21	0.1961 (3)	0.0160 (2)	0.6857 (2)	0.0419 (6)	
H21	0.247770	−0.052834	0.725248	0.049 (8)*	
C22	0.1244 (3)	0.0879 (2)	0.7316 (2)	0.0506 (7)	
H22	0.127846	0.067512	0.801438	0.053 (8)*	
C23	0.0476 (3)	0.1901 (2)	0.6733 (2)	0.0490 (7)	
H23	−0.002501	0.238136	0.704015	0.049 (8)*	
C24	0.0450 (3)	0.2211 (2)	0.5698 (2)	0.0427 (6)	
H24	−0.005800	0.290501	0.530513	0.048 (8)*	
C25	0.1175 (3)	0.1496 (2)	0.52394 (19)	0.0359 (6)	
H25	0.116296	0.171620	0.453750	0.037 (7)*	
C26	0.7137 (3)	0.1742 (2)	0.00625 (19)	0.0368 (6)	
C27	0.7700 (3)	0.2947 (2)	0.23968 (18)	0.0345 (6)	

### Atomic displacement parameters (Å^2^)

**Table:**

	*U*^11^	*U*^22^	*U*^33^	*U*^12^	*U*^13^	*U*^23^
Fe1	0.02404 (18)	0.02930 (18)	0.03075 (19)	−0.00346 (13)	−0.00173 (13)	−0.00939 (14)
S1	0.0808 (6)	0.0533 (5)	0.1010 (7)	−0.0154 (4)	0.0440 (5)	−0.0481 (5)
S2	0.0373 (4)	0.0653 (5)	0.0640 (5)	−0.0146 (3)	−0.0200 (3)	−0.0040 (4)
N1	0.0271 (10)	0.0298 (10)	0.0312 (10)	−0.0087 (8)	−0.0005 (8)	−0.0121 (9)
N2	0.0294 (11)	0.0304 (10)	0.0334 (11)	−0.0020 (8)	−0.0054 (8)	−0.0124 (9)
N3	0.0412 (12)	0.0357 (11)	0.0358 (12)	−0.0008 (9)	−0.0053 (9)	−0.0149 (9)
N4	0.0365 (12)	0.0361 (11)	0.0396 (12)	−0.0018 (9)	−0.0041 (9)	−0.0199 (10)
N5	0.0353 (11)	0.0284 (10)	0.0316 (11)	−0.0042 (8)	−0.0030 (8)	−0.0130 (9)
N6	0.0276 (10)	0.0269 (10)	0.0330 (11)	−0.0037 (8)	−0.0028 (8)	−0.0091 (9)
N7	0.0296 (11)	0.0307 (10)	0.0341 (11)	−0.0061 (8)	−0.0025 (8)	−0.0056 (9)
N8	0.0280 (10)	0.0274 (10)	0.0325 (11)	−0.0041 (8)	0.0008 (8)	−0.0068 (9)
N9	0.0371 (14)	0.0676 (16)	0.0621 (16)	0.0012 (11)	0.0028 (12)	−0.0361 (14)
N10	0.0295 (12)	0.0374 (12)	0.0464 (13)	−0.0031 (9)	−0.0078 (9)	−0.0122 (10)
C1	0.080 (2)	0.0589 (19)	0.0332 (15)	−0.0080 (17)	−0.0100 (15)	−0.0215 (15)
C2	0.0385 (15)	0.0481 (16)	0.0431 (16)	−0.0039 (12)	−0.0114 (12)	−0.0196 (13)
C3	0.0418 (14)	0.0362 (13)	0.0270 (12)	−0.0057 (11)	−0.0060 (10)	−0.0133 (11)
C4	0.0412 (15)	0.0341 (13)	0.0278 (12)	−0.0075 (11)	0.0019 (10)	−0.0104 (11)
C5	0.0321 (13)	0.0261 (12)	0.0337 (13)	−0.0073 (10)	−0.0063 (10)	−0.0087 (10)
C6	0.0257 (12)	0.0245 (11)	0.0373 (13)	−0.0031 (9)	−0.0070 (10)	−0.0108 (10)
C7	0.0357 (14)	0.0291 (13)	0.0426 (15)	0.0014 (10)	−0.0076 (11)	−0.0157 (11)
C8	0.0528 (18)	0.0564 (18)	0.0449 (16)	−0.0012 (14)	0.0061 (13)	−0.0287 (15)
C9	0.0437 (16)	0.0541 (17)	0.0522 (17)	0.0130 (13)	−0.0100 (13)	−0.0351 (15)
C10	0.070 (2)	0.067 (2)	0.065 (2)	−0.0053 (17)	−0.0127 (17)	−0.0413 (18)
C11	0.074 (3)	0.069 (2)	0.118 (3)	−0.004 (2)	−0.019 (2)	−0.055 (3)
C12	0.061 (2)	0.111 (3)	0.132 (4)	0.025 (2)	−0.039 (3)	−0.101 (3)
C13	0.066 (3)	0.143 (4)	0.090 (3)	0.034 (3)	−0.033 (2)	−0.089 (3)
C14	0.060 (2)	0.096 (3)	0.058 (2)	0.0226 (19)	−0.0177 (17)	−0.049 (2)
C15	0.0471 (16)	0.0363 (13)	0.0335 (13)	−0.0077 (11)	−0.0022 (11)	−0.0176 (11)
C16	0.0400 (14)	0.0351 (13)	0.0395 (14)	−0.0119 (11)	−0.0038 (11)	−0.0174 (12)
C17	0.0312 (13)	0.0275 (12)	0.0371 (13)	−0.0065 (10)	−0.0023 (10)	−0.0127 (10)
C18	0.0332 (14)	0.0358 (13)	0.0375 (14)	−0.0137 (11)	−0.0005 (11)	−0.0107 (11)
C19	0.0339 (14)	0.0301 (13)	0.0334 (13)	−0.0023 (10)	−0.0007 (10)	−0.0044 (11)
C20	0.0259 (12)	0.0291 (12)	0.0336 (13)	−0.0070 (9)	−0.0018 (10)	−0.0072 (10)
C21	0.0439 (16)	0.0387 (14)	0.0409 (15)	0.0017 (12)	−0.0129 (12)	−0.0116 (12)
C22	0.0627 (19)	0.0567 (18)	0.0413 (16)	−0.0051 (14)	−0.0105 (14)	−0.0265 (14)
C23	0.0525 (18)	0.0435 (16)	0.0619 (19)	−0.0059 (13)	−0.0020 (14)	−0.0317 (15)
C24	0.0394 (15)	0.0281 (13)	0.0569 (18)	−0.0023 (11)	−0.0001 (12)	−0.0122 (13)
C25	0.0329 (14)	0.0325 (13)	0.0342 (14)	−0.0055 (10)	0.0006 (10)	−0.0033 (11)
C26	0.0356 (14)	0.0337 (13)	0.0429 (15)	−0.0039 (11)	−0.0030 (12)	−0.0160 (12)
C27	0.0349 (15)	0.0296 (12)	0.0315 (13)	−0.0023 (10)	−0.0025 (10)	−0.0028 (10)

### Geometric parameters (Å, º)

**Table:**

Fe1—N1	2.1911 (19)	C5—C6	1.446 (3)
Fe1—N2	2.306 (2)	C6—C7	1.364 (3)
Fe1—N5	2.2618 (19)	C7—H7	0.9300
Fe1—N6	2.1817 (18)	C8—H8A	0.9700
Fe1—N9	2.088 (2)	C8—H8B	0.9700
Fe1—N10	2.088 (2)	C8—C9	1.511 (4)
S1—C26	1.620 (3)	C9—C10	1.370 (4)
S2—C27	1.621 (3)	C9—C14	1.383 (4)
N1—C4	1.460 (3)	C10—H10	0.9300
N1—C5	1.271 (3)	C10—C11	1.397 (4)
N2—N3	1.305 (3)	C11—H11	0.9300
N2—C6	1.361 (3)	C11—C12	1.376 (6)
N3—N4	1.355 (3)	C12—H12	0.9300
N4—C7	1.339 (3)	C12—C13	1.357 (6)
N4—C8	1.466 (3)	C13—H13	0.9300
N5—C15	1.464 (3)	C13—C14	1.373 (5)
N5—C16	1.267 (3)	C14—H14	0.9300
N6—N7	1.310 (3)	C15—H15A	0.9700
N6—C17	1.359 (3)	C15—H15B	0.9700
N7—N8	1.345 (2)	C16—H16	0.9300
N8—C18	1.338 (3)	C16—C17	1.447 (3)
N8—C19	1.459 (3)	C17—C18	1.362 (3)
N9—C26	1.147 (3)	C18—H18	0.9300
N10—C27	1.161 (3)	C19—H19A	0.9700
C1—H1A	0.9600	C19—H19B	0.9700
C1—H1B	0.9600	C19—C20	1.505 (3)
C1—H1C	0.9600	C20—C21	1.386 (3)
C1—C3	1.530 (3)	C20—C25	1.390 (3)
C2—H2A	0.9600	C21—H21	0.9300
C2—H2B	0.9600	C21—C22	1.380 (4)
C2—H2C	0.9600	C22—H22	0.9300
C2—C3	1.529 (3)	C22—C23	1.380 (4)
C3—C4	1.543 (3)	C23—H23	0.9300
C3—C15	1.530 (3)	C23—C24	1.374 (4)
C4—H4A	0.9700	C24—H24	0.9300
C4—H4B	0.9700	C24—C25	1.379 (4)
C5—H5	0.9300	C25—H25	0.9300
			
N1—Fe1—N2	72.65 (7)	N4—C7—H7	127.6
N1—Fe1—N5	77.61 (7)	C6—C7—H7	127.6
N5—Fe1—N2	107.15 (7)	N4—C8—H8A	108.4
N6—Fe1—N1	134.53 (7)	N4—C8—H8B	108.4
N6—Fe1—N2	82.74 (7)	N4—C8—C9	115.4 (2)
N6—Fe1—N5	73.95 (7)	H8A—C8—H8B	107.5
N9—Fe1—N1	94.52 (9)	C9—C8—H8A	108.4
N9—Fe1—N2	159.71 (8)	C9—C8—H8B	108.4
N9—Fe1—N5	84.55 (8)	C10—C9—C8	123.0 (3)
N9—Fe1—N6	116.89 (9)	C10—C9—C14	118.9 (3)
N10—Fe1—N1	116.78 (7)	C14—C9—C8	118.0 (3)
N10—Fe1—N2	84.55 (8)	C9—C10—H10	120.0
N10—Fe1—N5	164.17 (7)	C9—C10—C11	120.0 (3)
N10—Fe1—N6	97.64 (7)	C11—C10—H10	120.0
N10—Fe1—N9	87.58 (9)	C10—C11—H11	120.0
C4—N1—Fe1	121.79 (15)	C12—C11—C10	120.0 (4)
C5—N1—Fe1	119.40 (16)	C12—C11—H11	120.0
C5—N1—C4	117.8 (2)	C11—C12—H12	120.0
N3—N2—Fe1	137.20 (15)	C13—C12—C11	119.9 (4)
N3—N2—C6	109.88 (19)	C13—C12—H12	120.0
C6—N2—Fe1	111.96 (15)	C12—C13—H13	119.9
N2—N3—N4	106.06 (18)	C12—C13—C14	120.3 (4)
N3—N4—C8	119.0 (2)	C14—C13—H13	119.9
C7—N4—N3	111.3 (2)	C9—C14—H14	119.5
C7—N4—C8	129.7 (2)	C13—C14—C9	120.9 (4)
C15—N5—Fe1	125.38 (14)	C13—C14—H14	119.5
C16—N5—Fe1	115.78 (16)	N5—C15—C3	112.87 (19)
C16—N5—C15	118.8 (2)	N5—C15—H15A	109.0
N7—N6—Fe1	135.01 (15)	N5—C15—H15B	109.0
N7—N6—C17	109.86 (18)	C3—C15—H15A	109.0
C17—N6—Fe1	113.90 (14)	C3—C15—H15B	109.0
N6—N7—N8	106.19 (18)	H15A—C15—H15B	107.8
N7—N8—C19	119.86 (19)	N5—C16—H16	121.5
C18—N8—N7	111.16 (19)	N5—C16—C17	116.9 (2)
C18—N8—C19	128.89 (19)	C17—C16—H16	121.5
C26—N9—Fe1	176.7 (2)	N6—C17—C16	118.5 (2)
C27—N10—Fe1	165.1 (2)	N6—C17—C18	107.5 (2)
H1A—C1—H1B	109.5	C18—C17—C16	134.0 (2)
H1A—C1—H1C	109.5	N8—C18—C17	105.3 (2)
H1B—C1—H1C	109.5	N8—C18—H18	127.3
C3—C1—H1A	109.5	C17—C18—H18	127.3
C3—C1—H1B	109.5	N8—C19—H19A	109.0
C3—C1—H1C	109.5	N8—C19—H19B	109.0
H2A—C2—H2B	109.5	N8—C19—C20	113.00 (18)
H2A—C2—H2C	109.5	H19A—C19—H19B	107.8
H2B—C2—H2C	109.5	C20—C19—H19A	109.0
C3—C2—H2A	109.5	C20—C19—H19B	109.0
C3—C2—H2B	109.5	C21—C20—C19	118.8 (2)
C3—C2—H2C	109.5	C21—C20—C25	118.4 (2)
C1—C3—C4	106.8 (2)	C25—C20—C19	122.7 (2)
C2—C3—C1	109.2 (2)	C20—C21—H21	119.5
C2—C3—C4	111.5 (2)	C22—C21—C20	121.0 (2)
C2—C3—C15	110.2 (2)	C22—C21—H21	119.5
C15—C3—C1	107.8 (2)	C21—C22—H22	120.1
C15—C3—C4	111.2 (2)	C21—C22—C23	119.7 (3)
N1—C4—C3	111.39 (18)	C23—C22—H22	120.1
N1—C4—H4A	109.4	C22—C23—H23	120.0
N1—C4—H4B	109.4	C24—C23—C22	120.0 (3)
C3—C4—H4A	109.4	C24—C23—H23	120.0
C3—C4—H4B	109.4	C23—C24—H24	119.9
H4A—C4—H4B	108.0	C23—C24—C25	120.2 (2)
N1—C5—H5	121.1	C25—C24—H24	119.9
N1—C5—C6	117.7 (2)	C20—C25—H25	119.7
C6—C5—H5	121.1	C24—C25—C20	120.6 (2)
N2—C6—C5	117.2 (2)	C24—C25—H25	119.7
N2—C6—C7	107.9 (2)	N9—C26—S1	179.2 (3)
C7—C6—C5	134.9 (2)	N10—C27—S2	179.6 (3)
N4—C7—C6	104.9 (2)		
			
Fe1—N1—C4—C3	73.0 (2)	C1—C3—C15—N5	177.6 (2)
Fe1—N1—C5—C6	−0.8 (3)	C2—C3—C4—N1	55.2 (3)
Fe1—N2—N3—N4	167.35 (16)	C2—C3—C15—N5	−63.3 (3)
Fe1—N2—C6—C5	11.4 (2)	C4—N1—C5—C6	167.84 (19)
Fe1—N2—C6—C7	−171.13 (15)	C4—C3—C15—N5	60.8 (3)
Fe1—N5—C15—C3	−59.1 (3)	C5—N1—C4—C3	−95.4 (2)
Fe1—N5—C16—C17	−1.8 (3)	C5—C6—C7—N4	177.4 (2)
Fe1—N6—N7—N8	166.53 (16)	C6—N2—N3—N4	0.0 (2)
Fe1—N6—C17—C16	10.7 (3)	C7—N4—C8—C9	−79.0 (3)
Fe1—N6—C17—C18	−169.65 (16)	C8—N4—C7—C6	−178.2 (2)
N1—C5—C6—N2	−7.7 (3)	C8—C9—C10—C11	177.6 (3)
N1—C5—C6—C7	175.7 (2)	C8—C9—C14—C13	−176.8 (3)
N2—N3—N4—C7	0.4 (3)	C9—C10—C11—C12	−0.5 (5)
N2—N3—N4—C8	178.3 (2)	C10—C9—C14—C13	−0.3 (5)
N2—C6—C7—N4	0.6 (3)	C10—C11—C12—C13	−1.3 (6)
N3—N2—C6—C5	−177.83 (19)	C11—C12—C13—C14	2.3 (6)
N3—N2—C6—C7	−0.4 (3)	C12—C13—C14—C9	−1.5 (5)
N3—N4—C7—C6	−0.6 (3)	C14—C9—C10—C11	1.3 (5)
N3—N4—C8—C9	103.5 (3)	C15—N5—C16—C17	175.0 (2)
N4—C8—C9—C10	37.7 (4)	C15—C3—C4—N1	−68.1 (3)
N4—C8—C9—C14	−146.0 (3)	C16—N5—C15—C3	124.5 (2)
N5—C16—C17—N6	−6.0 (3)	C16—C17—C18—N8	179.7 (3)
N5—C16—C17—C18	174.4 (3)	C17—N6—N7—N8	0.4 (2)
N6—N7—N8—C18	−0.4 (3)	C18—N8—C19—C20	−102.2 (3)
N6—N7—N8—C19	−177.12 (19)	C19—N8—C18—C17	176.5 (2)
N6—C17—C18—N8	0.1 (3)	C19—C20—C21—C22	−175.6 (2)
N7—N6—C17—C16	180.0 (2)	C19—C20—C25—C24	175.1 (2)
N7—N6—C17—C18	−0.4 (3)	C20—C21—C22—C23	0.0 (4)
N7—N8—C18—C17	0.1 (3)	C21—C20—C25—C24	−1.9 (3)
N7—N8—C19—C20	74.0 (3)	C21—C22—C23—C24	−1.2 (4)
N8—C19—C20—C21	−166.2 (2)	C22—C23—C24—C25	0.8 (4)
N8—C19—C20—C25	16.8 (3)	C23—C24—C25—C20	0.8 (4)
C1—C3—C4—N1	174.4 (2)	C25—C20—C21—C22	1.5 (4)

### Hydrogen-bond geometry (Å, º)

*Cg* is the centroid of the C20–C25 ring.

**Table:**

*D*—H···*A*	*D*—H	H···*A*	*D*···*A*	*D*—H···*A*
C18—H18···*Cg*^i^	0.93	2.42	3.330 (3)	167
C19—H19*A*···N7^ii^	0.97	2.38	3.311 (3)	162
C21—H21···C27^ii^	0.93	2.89	3.603 (3)	134
C7—H7···S1^iii^	0.93	2.87	3.755 (3)	159
C4—H4*B*···N10^iii^	0.97	2.69	3.617 (3)	160
C4—H4*B*···C27^iii^	0.97	2.75	3.709 (3)	171

Symmetry codes: (i) −*x*, −*y*, −*z*+1; (ii) −*x*+1, −*y*, −*z*+1; (iii) −*x*+1, −*y*+1, −*z*.
